# Chromosome Territories in Hematological Malignancies

**DOI:** 10.3390/cells11081368

**Published:** 2022-04-17

**Authors:** Matheus Fabiao de Lima, Mateus de Oliveira Lisboa, Lucas E. L. Terceiro, Aline Rangel-Pozzo, Sabine Mai

**Affiliations:** 1 Department of Physiology and Pathophysiology, CancerCare Manitoba Research Institute, University of Manitoba, Winnipeg, MB R3E 0V9, Canada; matheus.fabiaodelima@umanitoba.ca; 2 Core for Cell Technology, School of Medicine, Pontifícia Universidade Católica do Paraná—PUCPR, Curitiba 80215-901, Brazil; mol.lisboa@gmail.com; 3 Department of Pathology, Max Rady College of Medicine, University of Manitoba, Winnipeg, MB R3E 3P5, Canada; evangel4@myumanitoba.ca

**Keywords:** multiple myeloma, nuclear organization, genome markers, leukemia, lymphoma, chromosome territories, nuclear architecture, differentiation

## Abstract

Chromosomes are organized in distinct nuclear areas designated as chromosome territories (CT). The structural formation of CT is a consequence of chromatin packaging and organization that ultimately affects cell function. Chromosome positioning can identify structural signatures of genomic organization, especially for diseases where changes in gene expression contribute to a given phenotype. The study of CT in hematological diseases revealed chromosome position as an important factor for specific chromosome translocations. In this review, we highlight the history of CT theory, current knowledge on possible clinical applications of CT analysis, and the impact of CT in the development of hematological neoplasia such as multiple myeloma, leukemia, and lymphomas. Accumulating data on nuclear architecture in cancer allow one to propose the three-dimensional nuclear genomic landscape as a novel cancer biomarker for the future.

## 1. Introduction

### Nuclear Architecture: Organization from Nucleotides to Chromosome Territories

The eukaryotic nuclei display a hierarchical chromatin organization that ranges from a single hydrogen bond (among complementary DNA bases—ATCG) to well-organized structures known as chromosome territories (CT) [1,2,3]. Higher levels of organization are required given the limited nuclear space in comparison to DNA size [1,4]. The first level of genome organization is represented by a simple nucleotide-nucleotide interaction (double DNA helix formation) [5]. This organization is necessary not only for the stability of the DNA molecule, but also for DNA folding [5,6]. The association of the double-stranded DNA with histone proteins constitutes the second level of chromatin organization, an octameric complex known as nucleosomes. The DNA-histone complex represents the 10 nm chromatin fiber known as “beads on a string” [7]. Small interactions between nucleosomes represent the third organizational structure of chromatin and may occur to generate the 30 nm chromatin fibers. It is worth mentioning that the visualization or even the existence of such 30 nm chromatin fibers remains the subject of investigation [8,9,10]; for a more detailed discussion about 30 nm fibers’ existence in different species, see [11]. Nevertheless, electron microscopy (EM) data also suggest the existence of a higher-level chromatin arrangement prior to mitosis or even in early stages of prophase, constituting what was called chromonema (chromatin fibers ranging from 200 to 250 nm) [12,13]. This chromatin arrangement was mainly observed in plant-derived cells where chromonema was described to be arranged helically [14]. However, according to a recent study, chromatin in mitotic chromosomes is organized as a series of linear loops emerging from a shared axis, non-affected through cell cycle phases [15].

Interactions of chromatin fibers and chromosome scaffold proteins (condensin I and II, topoisomerase II, as well as CTCF/cohesin complex), resulting in the formation of chromatin loops (ranging from 20 kbp to several Mbs in size), represent the fourth level of chromatin organization [16,17,18,19,20,21,22,23,24,25]. The CCCTC-Binding Factor (CTCF) associated with cohesin mediates the formation of chromatin loops through a process called loop extrusion [25,26,27,28]. This is essential not only for the sake of chromatin organization but also a rather key element in chromatin loop domain-mediated gene replication and chromatin interaction, as supported by many studies using the chromosome conformation capture (HI-C) technique [29,30,31,32]. The condensins and topoisomerase II are key proteins during chromosome condensation prior to cellular mitosis, regarded to keep the chromatin loop structure of chromosomes even after complete cell division, according to some studies [33,34,35]. The chromosome scaffold proteins are also involved in the process of chromatin organization constituted by several structures of chromatin loops surrounded by interchromatin spaces [1,36,37]. In this stage, the chromatin is organized into genomic domains known as chromatin domains (CD), the building blocks of the chromosome territories [36,38]. Ultimately, aggregation of chromatin domains into distinct nuclear spaces leads to the highest level of chromatin organization, the so-called chromosome territories [39,40,41]. This high level of genomic organization is maintained even during dynamic nuclear events, such as cell cycle and gene expression [42,43]. However, this well-balanced nuclear architecture can be modified for different diseases, including cancer [44,45,46]. In this review, we will discuss how changes in chromosome position can affect the cellular homeostasis in hematological cancers and how CT may serve as a future biomarker.

## 2. History of Chromosome Territories

**Figure 1:**
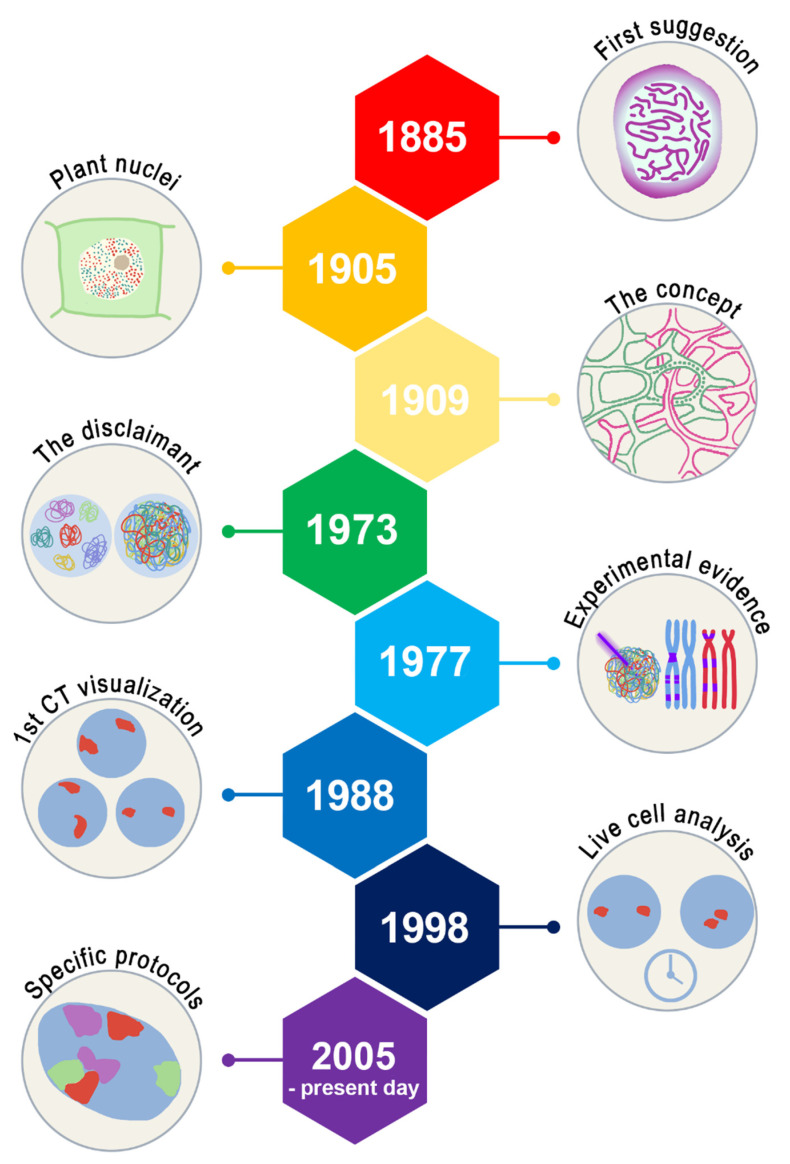
History of chromosome territories. In 1885, Carl Rabl alluded to what nowadays is termed chromosome territories. In 1905 and 1909, the chromosome territories concept was established based on studies in plant cells as well as horse roundworms and other organisms. In 1973, the chromosome territories were disclaimed by experiments using electron microscopy, marking the belief that chromatin would freely intermingle like a bowl of spaghetti. In 1977, the first concrete experimental evidence came to support the chromosome territories theory with Stack’s experiments. In 1988, the first visualization of a territorial-like organization of chromosomes was possible using the fluorescence in situ hybridization (FISH) technique. In 1998, the chromosome territories could be followed in vivo for a few minutes using live cell analysis (represented by the clock in the figure). Finally, with the association of chromosome probes, 3D-FISH protocols, confocal microscopy, and 3D image analysis, all chromosome territories could be studied in detail and in the same cell for the first time in 2005 [50]. For additional details and references, see text.

The term “chromosome territories” was first coined by Theodor Boveri (1909) in the 20th century. However, the idea of a territorial-like organization of chromosomes during interphase appeared as early as 1885, described by Carl Rabl, based on his experiments of cell division using *Salamandra maculata*. Rabl observed a polarized nuclear position of chromosomes at the beginning and at the end of mitosis, suggesting a preserved chromosome position during cell cycle phases [47]. In his studies, Boveri reached the same conclusion about the territorial organization of chromosomes [48]. Boveri followed individual chromosomes in horse’s roundworm throughout cell division. First, he noticed that the chromosomes assumed a specific nuclear position at the end of one mitosis (anaphase-telophase) and the beginning of the next one (prophase) [48]. Second, he observed that these chromosome territories could be composed of a network of chromatin bundles surrounded by interchromatin spaces, and third, he found that the chromosome territories and their neighborhoods were conserved during interphase. On a side note, Eduard Strasburger published a cartoon, in 1905, showing a territorial-like organization of chromosomes in the nuclei of plants, suggesting that the CT theory might be applicable to other than just animal cells (Figure 1) [49]. 

In the 1970s, with the advent of electron microscopy, new research data led to controversial observations. Wischnitzer (1973) argued that in an interphase state, the chromatin was intermingling freely inside of the cell nucleus, with no apparent individual pattern (bowl of spaghetti) [39,40,41,42,43,45,46,47,48,49,51]. Stack observed large patches of chromatin from previously fixed, air-dried, and Giemsa-stained Chinese hamster ovary cells (CHO), which Stack labeled as CTs. He also investigated the chromosomes during the cell cycle. His observation during mitosis revealed that chromosomes acquired a similar position at the beginning of prophase and at the ending of telophase, suggesting a conserved chromosome position (Stack, 1977). It is important to question whether Stack’s methods of sample preparation could have induced a territorial-like arrangement of the chromosomes [52]. 

Moreover, studies published by Cremer et al. (1979, 1984b) using Chinese hamster cells showed that, after nuclear damage inflicted by a micro laser beam (λ = 257 nm) in a small region of the nucleus, the region/DNA capable of recovering (pulse-labeled with ^3^H-thymidine), was restricted to a small number of chromosome regions [53,54]. These results indicated that chromatin was not freely intermingling inside the nucleus, as previously believed, but it was organized in territorial domains (according to the CT theory).

However, it was only in the 1980s that it was possible to perform the first visualization of the chromosome territories, with the development of in situ hybridization techniques, such as fluorescence in situ hybridization (FISH). Human hybrid cell lines were subjected to hybridization using DNA probes composed of biotin-labeled nucleotides or ^3^H- nucleotides followed by specific anti-biotin antibodies or BRL BluGene Nonradioactive Nucleic Acid Detection System [55,56]. This technique allowed chromosomes to be followed for a short period of time (in interphase cells) with visualization of chromosome territories in living cells [57,58]. A few years later, the dynamics of the chromosome territories through the cell cycle phase and the spatial arrangements of chromosomes with high resolution could be demonstrated through the association of 3D-FISH protocols, confocal microscopy, and 3D image reconstruction [58,59,60,61,62].

### 2.1. Chromosome Territories: Stablished Concepts 

**Figure 2:**
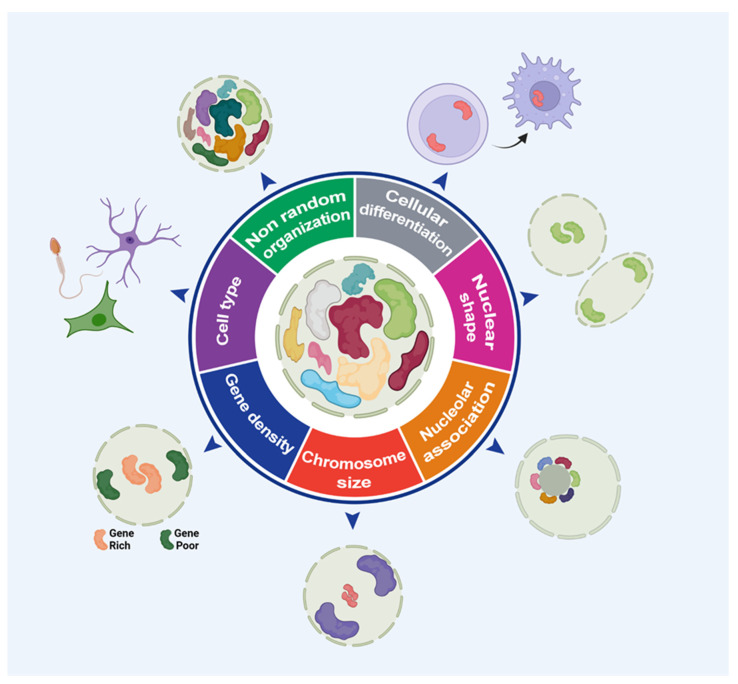
Chromosome territories: established concepts. Overview of chromosome organization regarding different proposed models of CT etiology. The non-random chromosome organization varies according to the cellular differentiation status, nuclear shape, nucleolar-associated chromosomes, chromosome size, gene density, and cell type. Chromosome spatial organization is emerging as an important mechanism of gene expression regulation and key determinant of cell fate. Image inspired by hallmarks of cancer, 2011 [72].

Chromosomes occupy specific positions, or territories, in an interphase nucleus [46,50] and possess a non-random distribution that varies according to cell type [63,64,65,66] (Figure 2). Different studies reported a relationship between chromosome distribution and gene density, with gene-rich chromosomes assuming an internal nuclear position while gene-poor chromosomes assume a peripheral localization [63,64,67,68,69] (Figure 2). This internal/central or peripheral nuclear organization of chromosomes has functional implications. The chromatin replication event and gene expression machinery are more concentrated in the center of the nucleus. Gene-rich chromosomes, commonly found in the nuclear center, are replicated first, while gene-poor chromosomes, associated with the nuclear periphery, are duplicated at the end of the S phase [63,70,71] (Figure 2). 

The territorial arrangements of chromosomes, preserved through evolution, enable compartmentalized gene expression [73,74,75,76]. Interestingly, active genes can be moved to the border of a CT or even to an extraterritorial region during the transcription process and return to their positions right after the transcription is complete [77,78,79]. Moreover, the attachment of chromatin to the nuclear lamina is a powerful cellular mechanism of gene repression [80,81,82,83]. Olfactory sensory neurons use the nuclear periphery as a repressive gene zone, required physiologically for smell perception [84,85,86]. In contrast, investigations of mammal rod cells revealed an inverted CT pattern: heterochromatin in the center and euchromatin in the periphery [87], suggesting that gene expression regulation mediated through association with the nuclear lamina is cell type specific, and in this case, essential for nocturnal vision [87]. Therefore, chromosome position goes beyond the mere organizational feature of the eukaryotic nucleus; it is also an important player in several processes related to cellular function and homeostasis.

Chromosomes may follow a size-rule distribution. Large chromosomes are localized at the periphery, while small chromosomes are found in the nuclear interior, regardless of possessing similar gene density composition [50,63,88,89] (Figure 2). The nuclear shape also affects chromosome positioning when comparing ellipsoid-shaped nuclei with round-shaped nuclei (Figure 2). For example, CT of chromosomes 3 and 11 in ellipsoid-shaped nuclei of fibroblasts exhibit CT 3 position towards the nuclear center and CT 11 towards the nuclear periphery, whereas round-shaped nuclei found in lymphocytes display CT 3 towards the nuclear periphery and CT 11 towards the nuclear center [90,91,92].

Some acrocentric chromosomes (CT 13, 14, 15, 21, and 22) were commonly observed in association with the nucleoli [89,93,94] (Figure 2). Such chromosomes contribute to the formation of the nucleolar organizing region (NOR) through the association of their *p*-arms (which mostly contain sequences of ribosomal DNA) with the nucleoli [89,93,94]. NOR-CT is essential not only for ribosome synthesis, but also to provide organizational clues for other homologous chromosome territories [95]. Moreover, some studies in fibroblast nuclei revealed an increased frequency of chromosome association related to the NOR-CT, up to 82% (as shown for the pairwise CT 15–21), which may influence the occurrence of chromosome translocations involving the CT 15–21, observed in myeloid disorders such as acute myeloid leukemia [89,96]. Furthermore, some physiological conditions (cellular quiescency, cell cycle, and cell differentiation) have also been shown to alter chromosome positioning in a transient or permanent fashion (Figure 2). This suggests a possible role of CT dynamics upon different cellular processes [66,97,98,99,100].

### 2.2. Cell Differentiation and Changes in CT of Hematological Cells

The three-dimensional organization of chromatin plays a crucial role in cell differentiation by modulating the transcription process, DNA replication, and cell division [51]. CT positions are cell type-specific and change during cellular differentiation [101]. However, there are some exceptions for specific chromosomal regions, in which it is possible to see a similar organization between different cell types. Parada et al. (2004) reported that higher CTs similarities were found in cell types presenting common differentiation pathways. This pattern was also observed in lymphoblast and myoblast cells, in which only the position of chromosome 5 differed between the two cell types [101]. 

Regarding CT changes during differentiation, Bártová et al. (2000) showed that the HL-60 cell line, after granulocytic differentiation induced by dimethyl sulfoxide (DMSO) or all-trans-retinoic acid (ATRA), presented a more peripherical position of *ABL* and *BCR* genes. In addition, the distances of the homologous *ABL-ABL* and *BCR-BCR* genes increased in comparison to the promyelocytic state. Interestingly, after granulocytic differentiation, *c-MYC* was frequently more distant from its homologous partner and the nuclear center. Furthermore, in HL-60 cells treated with phorbol esters (PMA) to induce monocytic differentiation, the *ABL* and *BCR* genes were shifted closer to each other in the nuclear center [102].

Kosak et al. (2002) showed that the immunoglobulin (*Ig*) loci were located at the nuclear periphery in hematopoietic progenitors and pro-T cells but were centrally positioned in pro-B nuclei. This observation supports the hypothesis that subnuclear compartmentalization of immunoglobulin loci during lymphocyte development represents a mechanism of transcription regulation and recombination during differentiation [103]. Lomiento et al. (2018) investigated differences in chromosome positioning between hematopoietic stem cells (CD34^+^) and myeloid precursor cells (CD14^−^). Positions of chromosomes 8 and 9 were remarkably different between the two cell types. Although chromosome 8 was very peripheral in both cell types, it was closer to the nuclear border in the precursor cells. On the other hand, chromosome 9 was localized more internally in CD34^+^ and CD14^−^ cells but was more peripherally located in the myeloid precursors. Furthermore, the inter-homolog distances were consistently shorter in the myeloid precursor for chromosomes 6, 9, and 15, and chromosomal volumes of chromosomes 6 and 15 were significantly different between CD34^+^ and CD14^−^ cells [104]. The role of CTs in myeloid differentiation was also supported by observations of higher-order chromatin organization in active and inactive nuclear compartments. Hübner et al. (2015) demonstrated that during myeloid differentiation, there were significant differences in higher-order chromatin arrangements, nuclear localization, occupation of the interchromatin compartment, and the distribution of nuclear pores. This study also supports the model in which the nucleus has two main nuclear compartments, active and inactive nuclear compartments [105].

Evidence for general changes in CTs comes from studies evaluating the spatial distribution of centromeres in interphase nuclei during differentiation. This was demonstrated during T-cell, monocytic, granulocytic, and myeloid differentiation [106,107]. The arrangement of centromeres in interphase is stable, non-random, and cell-type specific. As expected, these changes in centromeres were frequently followed by changes in CTs. Chaly and Munro (1996) showed the repositioning of the centromeres to the nuclear periphery during myogenesis in mouse cells [108]. Kim et al. (2004) verified the spatial genome organization in differentiating mouse T-cells. Centromeres were moved to a preferentially peripheral position in the CD4^+^ and CD8^+^ differentiated cells [106]. Chromosome 6 changed its position during the transition of double-positive (lymphoid progenitor cells expressing both CD4 and CD8) to CD4^+^ and CD8^+^ cells [106]. These results are valuable in the context of lymphomagenesis since double-positive CD4^+^/CD8^+^ cells comprise a commonly found population in nodular lymphocyte predominant Hodgkin lymphoma (NLPHL), a B-cell neoplasm [109].

### 2.3. Clinical Applications 

The association between gene expression patterns and chromatin conformation signatures has emerged as a source of disease diagnosis [110]. Considering the small patient sample (interphase nuclei) required for examination and the fact that fluorescent imaging is already in clinical use, CT investigation arises as an important tool with the potential for applications in clinical settings. Chromosome positions in normal human cells could be used as a comparative point to identify pathological conditions, such as male infertility disorders that lack a good prognosis. Idiopathic male infertility displays altered chromosome organization as well as increased chromocenter numbers when compared to fertile men, leading to gamete fusion defects and consequently to male infertility [44,45,111,112,113]. Furthermore, it is known that cancer cells often display altered chromosome positions when compared to their normal counterparts [66,90,114,115,116]. Such altered positions could favor translocations between chromosomes from different CTs or induce changes in gene expression that leads to cancer progression [117,118,119,120,121]. In fact, CT investigations have been of great use to distinguish between non-malignant and malignant cervical squamous carcinoma cells by analyzing the position of CT 18, which displays an altered position toward the nuclear periphery in BCL2 positive carcinoma cells compared to CT 18 internal position observed in non-malignant squamous epithelium cells [120,122].

## 3. Chromosome Territories in Hematological Cancers 

### 3.1. Multiple Myeloma

The malignant transformation process is preceded by key alterations of DNA content or structure [66,90,114,115,116,123,124,125,126]. Several genomic alterations, such as DNA-damage-induced events, chromosome abnormalities, and epigenetic modifications, contribute to the high genomic instability observed in cancer [124,127,128,129]. Multiple Myeloma (MM) is an incurable disease of plasma cells characterized by the accumulation of aberrant cells in the bone marrow and secretion of immunoglobulin called M protein [130,131]. MM is the latest stage of a progressive disease preceded by two precursor asymptomatic stages (monoclonal gammopathy of unknown significance, MGUS, and smoldering multiple myeloma, SMM) [132]. The progression from MGUS to SMM and, ultimately, to MM is associated with numerical and structural chromosomal alterations, such as gains or losses of chromosomes and chromosome translocations, which may promote disease progression [133,134,135]. MM can be defined as high-risk based on the presence of genomic abnormalities, such as aneuploidies (gains or losses) in chromosomes 3, 5, 7, 9, 11, 15, 19, and 21, as well as translocation events that mostly comprise the immunoglobulin heavy-chain (*IGH*) locus, placed on chromosome 14 [136,137]. 

Studies using interphase nuclei of normal B-lymphocytes revealed a neighborhood relationship of chromosomes involved in MM translocations [119,138,139]. This chromosome proximity increases the probability of translocation events, which may explain why MM displays a diverse range of different translocations [136,138,139]. Balajee et al. (2018) showed that the frequency of chromosome translocation after irradiation (X-ray and neutrons) of B-lymphocytes was dependent on chromosome proximity [139]. Other studies performed in normal lymphocytes showed a similar relationship in which CT neighborhood arrangements would facilitate CT intermingling, especially in regions where translocation events are often reported [140]. Therefore, CT proximity would facilitate chromatin exchange events to occur [117], which could explain the occurrence of rarer translocations in MM patients, such as the ones involving the immunoglobulin lambda-chain (*Igλ*) and the MYC gene (poor prognosis molecular marker for MM) [136,139].

**Figure 3:**
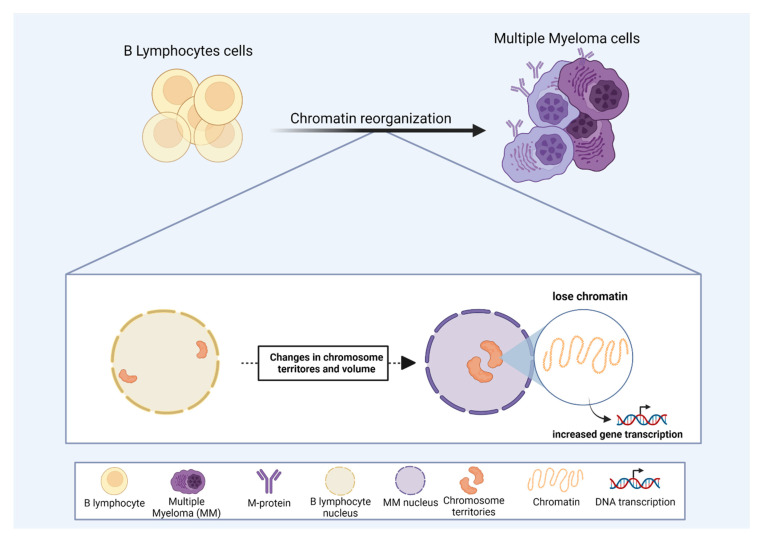
The role of chromosome territories in multiple myeloma (MM). Correlation of chromosome position and chromatin state as drivers of the malignant progression in MM. For details, please see Section 3.1. In MM, some chromosomes display altered positions, towards the nuclear center, compared to regular B lymphocytes. Some chromosome territory volumes also increase in MM, and changes in nuclear organization may favor MM progression by modulating gene expression.

One of the key regulators of gene expression is the chromatin state. The availability of chromatin sites to the transcriptional machinery can dictate gene expression [141,142]. In MM, recent studies have shown an association between chromatin status and altered gene expression [136,143,144]. Moreover, CT volumes increase in MM compared to normal B-lymphocytes [145] (Figure 3). This observation supports the idea that an open chromatin state in MM would facilitate the expression of key genes involved in MM development and progression [141,145]. The analysis of chromatin structure, using 3D Structured Illumination Microscopy in MGUS and MM derived patient samples, revealed that MM has a less condensed and an open chromatin state compared to normal B-lymphocytes, even in the precursor stage of MGUS [146], suggesting that chromatin state could be used to identify MM in early stages of the disease development.

Chromatin localization inside the nucleus can also modulate gene expression in MM. In MM-derived patient samples, the territories occupied by chromosomes 4, 9, 11, 14, and 18 are internally located compared to normal B-lymphocytes [145] (Figure 3). These altered CT positions associated with an open chromatin state might play an important role in deregulating gene expression, ultimately leading to MM progression (Figure 3). According to this concept, Broyl et al. (2010) and Chen et al. (2021) reported that several genes located on chromosomes 4, 9, 11, and 18 were up-regulated in MM-derived patient samples [147,148]. These studies provide insight that a high-level chromatin regulation could be critical for MM progression and highlight chromosome position and chromatin state as important prospective targets for MM therapeutical strategies.

### 3.2. Acute Myeloid Leukemia and Secondary Leukemia

Acute Myeloid Leukemia (AML) accounts for the majority of cases of secondary leukemias [149]. The most common chromosomal abnormality associated with AML is *t* (8;21) (q22;q22.1) [150], where up to 20% of these cases are secondary AML. This translocation leads to a novel chimeric gene *RUNX1*-*RUNX1T1* on chromosome 8. When not translocated, *RUNX1* and *RUNX1T1* do not share the same CT [151]. Rubtsov et al. (2008) showed that the treatment of primary embryonic normal human male fibroblasts with the Topoisomerase II (Topo II) poison etoposide leads to a reposition of the *RUNX1T1* gene. After Topo II treatment, *RUNX1* and *RUNX1T1* were found in closer proximity [151]. The authors did not observe a direct contact between the two genes in fibroblasts after etoposide treatment. Only human lymphoid cells (Jurkat cells) displayed a juxtaposition of *RUNX1* and *RUNX1T1* after the treatment, suggesting a cell type-specific relationship [152].

The mixed-lineage leukemia gene (*MLL*) is also frequently rearranged in secondary leukemias [153]. The breakpoint cluster region of *MLL* has a Topo II cleavage site [154,155]. Glukhov et al. (2013), after treating human lymphoid cells with etoposide, observed many cleavage sites of the *MLL* gene (~17% of nuclei). They showed that ~9% of these broken *MLL* alleles were repositioned outside their CT [156]. *MLL* can be translocated with more than 40 different partners [157,158]. Gué et al. (2006) measured the relative positions of *MLL* to *AF4* and ENL genes (a commonly involved gene and a less frequent gene in MLL translocations, respectively). Interestingly, *MLL* and *ENL* genes were closer to each other in comparison to *MLL* and *AF4* [159,160]. The “breakage first” model states that breaks formed at distant locations could scan for potential partners and move to produce translocations, which could explain their observation. There are many models contraposing the “breakage first” model; among them, figures the “contact-first” model. In this model, translocations preferentially occur between chromosomes that are in close spatial proximity, a hypothesis based on the observation that chromosomes are not randomly distributed in the interphase nuclei [160,161,162].

### 3.3. Radiation Effects on CT in Hematological Malignancies

Ionizing radiation is known to be one of the main causes of genetic instability [163]. Several studies demonstrated that chromosomal proximity plays a key role in translocations arising after irradiation [164]. Lukásová et al. (1997) compared *ABL* and *BCR* gene nuclear localizations in bone marrow cells from patients with chronic myeloid leukemia and control donors. After irradiation, both genes were shifted to the central area of the nucleus in approximately 15% of the cells [165]. Kozubek et al. (1997) also showed in lymphocytes exposed to radiation that *BCR* and *ABL* genes are shifted to the nuclear center in closer proximity. Bártová et al. (2000) also showed increased proximity of *ABL* and *BCR* in lymphocytes after irradiation. Interestingly, closer proximity was found between *c-MYC* and *IGH* genes in approximately 8% of the lymphocytes. Those two genes are involved in the *t* (8;14), a translocation present in 98% of Burkitt’s lymphoma cases [166,167]. In irradiated lymphocytes, *c-MYC* is shifted closer to the nuclear center. However, after 24 h of radiation exposure, *c-MYC* returns to its original territory [102]. 

Cafourková et al. (2001) also irradiated normal lymphocytes to verify CT alterations among 11 selected chromosomes. They observed many CT interactions after irradiation where the interactions were associated with chromosome size, as expected due to the increased probability of CT contact increases for larger chromosomes [168]. Similarly, Boei et al. (2006), using FISH, observed alterations in chromosomes 1, 4, 18, and 19 of lymphocytes exposed to ionizing radiation. They also found an association between chromosome size and CT distribution. The frequency of CT changes was associated with the density of ionizing radiation, presenting higher frequencies with densely ionizing radiation than with the sparse one [169]. Anderson et al. (2002) reported that complex chromosome aberrations (chromosomal exchanges involving three or more breaks in two or more chromosomes) arise in peripheral blood lymphocytes after exposure to high-linear energy transfer (LET) α-particle radiation [170]. Later, in 2006, they analyzed the formation of complex chromosomal abnormalities and reconstructed their probable origin. This model proposed by Anderson et al. (2006) suggests that in the individual high-LET α-particle-induced complex chromosomal abnormalities arise from the misrepair of damaged chromatin in single physical locations. The complexity is influenced by the number of CTs that are affected by alpha particles, which corroborates previous studies with irradiation. Due to their size, chromosome q-arms are more prone to translocations with different chromosomes [170,171]. Recently, Balajee et al. (2018) used multicolor FISH (m-FISH) to investigate CTs in interphase nuclei of lymphocytes and B-lymphoblastoid cells [139]. They analyzed cells before and after exposure to ionizing radiation using metaphase chromosome analysis. Up to 50% of the ionizing radiation-induced translocations were associated with the proximity of pre-existing CTs in both cell lines. Chromosome conformation capture (Hi-C) was used to measure the frequencies of interactions between different chromosomes and gene loci, and it highly correlated with the findings using FISH. There were also interactions between loci of the genes *BCR* and *ABL* in lymphoblastoid cells, which increased after exposure to X-ray. However, in the fibroblasts, this association was not observed [139]. Collectively, these studies provide a new layer of knowledge on the explanations for why some translocations are more likely to occur after exposure to radiation, one of the most studied factors known to increase the risk of hematological malignancies.

### 3.4. Lymphomas 

Hodgkin’s lymphoma is a disorder characterized by the presence of mono-nucleated Hodgkin cells and bi- to multi-nucleated Reed–Sternberg cells [172,173]. Guffei et al. (2010) demonstrated that chromosomes 9 and 22 have altered CTs in both mono- or multi-nucleated cells [174]. These data are consistent with previous results showing that the transition from Hodgkin to Reed–Sternberg cells is marked by changes in the three-dimensional nuclear organization of telomeres [175]. The nuclear telomeric architecture of Hodgkin’s and Reed–Sternberg cells was significantly different at diagnosis for cases with recurring/relapsed disease when compared to the non-relapsed group [176]. These data on Hodgkin’s lymphoma imply that changes in nuclear architecture, including CTs, are a key factor for the occurrence of chromosomal translocations and nuclear genome remodeling found in this disorder.

Roix et al. (2003) reported that the genes frequently translocated in B-cell lymphomas are positioned at a closer distance in normal human B cells. *MYC* was found in significantly closer proximity with *IGH* and *IGL* but distant from its rare translocation partner *IGK*. Furthermore, normal fibroblasts, when compared to lymphocytes, present longer distances between *MYC* and *IGH* loci. This provides strong evidence for the role of cell-specific CT proximity since translocations involving these two genes do not occur in cell types other than B cells [120]. Furthermore, several factors are involved in the origin of lymphomas, and there is a significant association with several types of viral infection. Patients infected by the human immunodeficiency virus (HIV) present an increased risk of developing lymphoma. Burkitt lymphoma (BL) is one of the most predominant lymphomas associated with HIV infection. Most cases of BL have the *MYC-IGH* translocation. Interestingly, Germini et al. (2017) showed that the HIV Tat protein, a key factor in the HIV pathogenesis, when injected into circulating B-cells, generates DNA damage and changes in *MYC* gene CT. In this context, *MYC* moves to the nuclear center, colocalizing with the *IGH* (10-fold when compared to controls) [177].

Anaplastic large cell lymphoma (ALCL) is a non-Hodgkin’s lymphoma of cells expressing CD30. The translocation *t* (2; 5) (p23; q35) is found in approximately half of the ALCL cases [178]. Mathas et al. (2009) studied an ALCL cell line without this translocation, and they visualized close proximity between the two chromosomal regions involved in the t(2;5) (p23;q35). In order to further investigate the role of close proximity in facilitating translocation between these two chromosomal regions, the authors induced DSBs in the negative ALCL cells. The *t* (2;5) (p23; q35) was found in these cells after DNA repair [179].

Klein et al. (2011), investigating the nature of chromosomal rearrangements in mouse B lymphocytes, demonstrated that proximity between DSBs, transcriptional activity, and CTs were key factors of rearrangements. One part of the rearrangements was associated with CT, in which intra-chromosomal joining was more common than trans-chromosomal rearrangements after DSB formation. Remarkably, the rearrangements were preferentially found at the transcription start sites of actively transcribed genes [119].

CTs in human lymphocytes can interact more than previously anticipated. Recently, Steininger et al. (2018) applied the chromosome conformation capture (Hi-C), a technique used for the investigation of genome-wide chromatin interactions in a T-cell lymphoma cell line. They found higher probabilities of interaction between chromosomal segments than an earlier report [180]. Branco and Pombo (2006) used cryo-FISH (a FISH method using cryosections of approximately 150 nm thick of sucrose-embedded fixed cells or tissues) in human lymphocytes and reported that approximately 40% of each chromosome intermingled with the rest of the genome. A highly significant correlation was found between the extension of CT intermingling and the frequency of translocations in lymphocytes (*p* < 0.0001). It was also estimated that approximately 19% of the nuclear volume is composed of intermingling regions [117]. Tavares-Cadete et al. (2020) have shown different results, indicating that contact/entanglement of chromosomes and chromosomal domains are not so frequent. However, in this study, they used different cells (HeLa S3 cell line) [181]. 

There was a significant achievement in demonstrating that the spatial proximity of chromosomes increases the probability of translocations in hematological malignancies such as myeloma, leukemia, and lymphoma. However, only a few genes and chromosomes were investigated; a significant part of the studies was not confirmed later or were conducted using cell lines. There are still many potential genes involved in translocations of these diseases, and this offers a wide field to be explored. This field of study has gained remarkable new possibilities since new genome-wide/single-cell techniques are being tested. The biogenesis of chromosomal translocations is a highly complex phenomenon. Although differentiation, exposure to radiation, and cytotoxic drugs were associated with changes in CTs, which elevates the risk of chromosomal translocations, many other factors predisposing these alterations could also be studied in the context of CTs. Due to the complexity of the mechanisms behind the occurrence of translocation, it is essential to mention that although CTs are clearly associated with specific chromosomal abnormalities, many others still lack explanations. In this sense, CT comprises a critical candidate to explain such an important cancer hallmark. 

## 4. Conclusions

Chromosomes are known to assume non-random locations and neighborhood positions in the nucleus. DNA breakage, dynamics of chromosome movement, and chromosome positioning play important roles in this event, enabling tumor-associated translocation events to occur. As a result, neighboring chromosomes are commonly involved in translocations. Due to clonal evolution and divergence, new neighborhood chromosome relationships are likely to be established, resulting in new translocations and rearrangements.

It is clear that chromosome territories should be considered a key element in the pathogenesis of hematological malignancies. In fact, the observation of changes in chromosome positions from pre-malignant to malignant stages may be predictive of adverse chromosome associations that will trigger cancer development and/or evolution. Furthermore, changes in nuclear architecture and chromosome positions could be useful in the clinic to identify cancers at risk of progression, especially for diseases that lack specific biomarkers. However, more studies are needed to understand the pathways behind CT alteration in tumors as well as possible therapeutical approaches that target and modulate genome architecture. 

Chromosome position analysis in hematological malignancies has revealed altered genome organization in cancer cells compared to their normal cell counterparts. However, most of the studies focused on the mere description of the changes in positioning rather than showing the direct relationship between chromosome movement (to nuclear periphery to nuclear center or vise-versa) and the transcriptional activation or downregulation of genes located in the altered topological region. New techniques such as MERFISH and StarMAP, which combine chromosome position, gene transcription, and protein translation analysis, could provide a better overview of the precise events that occur at the molecular level [182,183,184,185,186]. The combination of FISH with Hi-C analysis could also provide an idea of chromosome associations promoted by malignant transformation. CT analysis is a single-cell technology. It provides an additional layer of information in another dimension to decipher the functional states of clonal subpopulations. Examining tumors this way, it will be possible to have a perspective of how cancer might react to treatment [187].

## Data Availability

No new data were created or analyzed in this study. Data sharing is not applicable to this article.
